# Exploring the Biocontrol Efficacy of *Trichoderma* spp. against *Rigidoporus microporus*, the Causal Agent of White Root Rot Disease in Rubber Trees (*Hevea brasiliensis*)

**DOI:** 10.3390/plants12051066

**Published:** 2023-02-27

**Authors:** Wen Ze Go, Kit Ling Chin, Paik San H’ng, Mui Yun Wong, Chuan Li Lee, Pui San Khoo

**Affiliations:** 1 Department of Wood and Fiber Industries, Faculty of Forestry and Environment, Universiti Putra Malaysia, Serdang 43400, Selangor, Malaysia; 2 Institute of Tropical Forestry and Forest Product, Universiti Putra Malaysia, Serdang 43400, Selangor, Malaysia; 3 Department of Plant Protection, Faculty of Agriculture, Universiti Putra Malaysia, Serdang 43400, Selangor, Malaysia; 4 Institute of Plantation Studies, Universiti Putra Malaysia, Serdang 43400, Selangor, Malaysia; 5 Centre for Advanced Composite Materials, Universiti Teknologi Malaysia, Johor Bahru 81310, Johor, Malaysia

**Keywords:** white root rot, *Rigidoporus microporus*, Trichoderma spp., biocontrol agent, rubber tree, dual culture, nursery trial

## Abstract

*Rigidoporus microporus,* which causes white root rot disease (WRD) in *Hevea brasiliensis,* is a looming threat to rubber plantation in Malaysia. The current study was conducted to determine and evaluate the efficiency of fungal antagonists (Ascomycota) against *R. microporus* in rubber trees under laboratory and nursery conditions. A total of 35 fungal isolates established from the rubber tree rhizosphere soil were assessed for their antagonism against *R. microporus* by the dual culture technique. *Trichoderma* isolates can inhibit the radial growth of *R. microporus* by 75% or more in the dual culture test. Strains of *T. asperellum*, *T. koningiopsis, T. spirale,* and *T. reesei* were selected to assess the metabolites involved in their antifungal activity. Results indicated that *T. asperellum* exhibited an inhibitory effect against *R. microporus* in both volatile and non-volatile metabolite tests. All *Trichoderma* isolates were then tested for their ability in producing hydrolytic enzymes such as chitinase, cellulase and glucanase, indole acetic acid (IAA), siderophores production, and phosphate solubilization. From the positive results of the biochemical assays, *T. asperellum* and *T. spirale* were selected as the biocontrol candidates to be further tested in vivo against *R. microporus*. The nursery assessments revealed that rubber tree clone RRIM600 pretreated with only *T. asperellum* or with the combination of *T. asperellum* and *T. spirale* was able to reduce the disease severity index (DSI) and exert higher suppression of *R. microporus* compared to other pretreated samples, with the average DSI below 30%. Collectively, the present study demonstrates that *T. asperellum* represents a potential biocontrol agent that should be further explored to control *R. microporus* infection on rubber trees.

## 1. Introduction 

In Malaysia, rubber (*Hevea brasiliensis*) leaves, stems, and roots are afflicted by a great number of diseases associated with fungal pathogens. Among these fungal infections, *Rigidoporus microporus,* which causes white root rot disease (WRD), is reported to be the most serious, as it often fatal to the tree and brings a severe reduction of latex production and economic loss [1,2,3]. WRD has caused a major loss of yield of up to 50% in old plantations with a great impact on the investors of rubber tree farming [4]. 

The white root rot pathogen, *R. microporus,* causes severe damage to the woody tissues of *H. brasiliensis,* which results in substantial death of the trees and sometimes in the loss of a whole stand [5]. This soil-borne pathogen can survive for years in the field by creating white mycelia that can stick to the surface of the root. The ability of mycelia to develop several meters long and spread through the soil infecting the neighboring healthy trees has been the main concern to rubber growers over the past decades [6,7].

*R. microporus* attacks plants at different stages. It appears in young rubber plantations as early as two years old plants and also in five- or six-years old plants, not limited to old rubber plantation trees which are also suffering from this disease [8]. Once the rubber tree is infected by the pathogen, the mycelium or white rhizomorphs of *R. microporus* can be seen attaching to the roots when exposed. As the disease infection progresses, the leaves show general discoloration, proceeded by premature flowering and fruiting of the trees. In the advanced stage, a large, firm, semi-fleshy, often tier-up, and brownish-orange bracket forms on the collar of infected rubber trees. The formations of the sporocarp normally appear only after the trees have been dead for a while [1]. 

For the past 20 years, higher success in controlling the WRD disease is achieved through the use of fungicides with different active ingredients such as triadimenol, triadimefon, propiconazole, terbuconazole, hexaconazole, and myclobutanil [9,10]. Utilizing chemical control is not only uneconomical, but it often does not display the much-needed efficacy against soil pathogens. The continuous usage of chemical control approaches fosters the development of resistant pathogens [11] while also destructively affecting the quality of food resources and the environment. Furthermore, agrochemical applications, particularly urea, fungicides, and herbicides, under extensive usage are likely to boost populations of root rot pathogens in field soils [12,13,14]. The search for a suitable strain of biological control agents (BCAs) with greater biocontrol activities is necessary for alternative strategies against *R. microporus,* as the cultural and chemical practices used by the farmers are less practical. 

BCAs, as a sustainable alternative to chemical control for plant diseases, are a popular research topic in this modern agricultural practice [3,15,16]. BCAs that are most extensively used belong to the genus *Trichoderma* for fungi and *Pseudomonas* and *Bacillus* for bacteria [17,18]. Numerous species of non-pathogenic rhizosphere fungi have been reported to be capable to control WRD in rubber trees caused by *R. microporus* in Thailand, Sri Lanka, Indonesia, and Nigeria [4,5,19,20,21]. *Trichoderma* spp. (division: ascomycota), a non-pathogenic rhizosphere fungus, has been receiving great attention in biological control research in the tropics [22,23,24]. *T. harzianum*, *T. hamatum*, *T. virens*, *T. atroviridis,* and *T. viride* are among the *Trichoderma* species which have been reported to control plant fungal diseases in Thailand and Indonesia [25,26].

In vitro screenings of antagonists have been widely used to select potential BCAs and elucidate their biocontrol mechanisms. There are numerous modes of action have been reported to be applied by *Trichoderma* spp. against plant pathogens. Generally, mechanisms comprised the biocontrol activities of fungal antagonists are presented in two ways: (i) the direct interaction (produce toxic compounds or antibiotics, synthesis of hydrolytic enzymes, and competition) and (ii) indirect interaction (induce resistance in the host plant) [27,28]. It was suggested by Suryanto et al. [21] that the application of BCAs prior to the pathogen infection will give a better result whereby the BCAs provide protective rather than curative treatment. 

In this study, selected *Trichoderma* isolates were tested for their efficacy as potential BCAs against *R. microporus*. Antagonistic efficiency was evaluated as in vitro production of volatile and non-volatile substances, hydrolytic enzymes (chitinase, cellulase, and glucanase), and plant growth-promoting traits, e.g., indole acetic acid (IAA), siderophores and phosphate solubilization. Nursery assessments were also conducted to provide insight into the natural interactions of host–pathogen–environment to ensure its effective use as a BCA in rubber plantations.

## 2. Materials and Methods

### 2.1. Pathogen and Potential Antagonists 

The pure isolate of *R. microporus* RL21 (accession number MN103602) was obtained from the Laboratory of Crop Improvement and Protection Unit, Rubber Research Institute of Malaysia (RRIM), and the virulence of this isolate against the rubber tree clone RRIM600 was published by Go et al. [29].

Searching for potential antagonistic fungi was performed through the collection of soil samples from the rhizosphere of healthy and WRD-infected rubber trees located in the Hevea Germplasm, Field 23, the Rubber Research Institute of Malaysia (RRIM), Sungai Buloh. Soil samples were collected from holes dug around healthy and WRD-infected rubber trees. The isolation of the soil-borne fungi was performed via the soil dilution plating method performed on the potato dextrose agar (PDA) (DifcoTM, Detroit, MI, USA), as described by Go et al. [30]. A serial dilution of the soil was performed for up to 10^−4^ using 1 g of soil added into 9 mL of distilled water. A total of 200 μL of the solution from the serial dilutions of 10^−3^ and 10^−4^ was spread onto PDA plates. Each dilution consisted of three replicates. The Petri dishes were placed in the incubator at 28 ± 2 °C for one week. Single spore isolation was performed on new plates of PDA to obtain a pure culture of the fungi.

For the DNA extraction, a fungal mass of approximately 100 mg was used. After the one-week incubation period, a fine sterilized spatula was used to scrape the actively growing fungal mass from the culture plate. The genomic DNA was extracted according to the method described by Lin et al. [31], with minor modifications. The primers ITS1 (5′-TCCGTAGGTGAACCTGCGG-3′) and ITS4 (5′-TCCTCCGCTTATTGAT ATGC-3′) were used for amplifying the ITS region [32]. The reaction system contained 12.5 µL of Taq Polymerase (1st BASE Biochemicals), 1.0 µL of template DNA, 1.0 µL of each forward and reverse primer, and 9.5 µL distilled water to a final volume of 25 µL. The amplification was carried out using Vapoprotect^®^ (Eppendorf, Hamburg, Germany) with the following protocol of cycling parameters: 98 °C for 2 min, followed by 25 cycles of 98 °C for 15 s, tempering at 60 °C for 30 s, extension at 72 °C for 30 s, and the final extension at 72 °C for 10 min. A total of 1% (*w*/*v*) agarose gel with FloroSafe DNA gel dye (1st BASE) was used to electrophorese the PCR products. At 70 V, electrophoresis was performed for 45 min and DNA fragments were visualized under a UV Transilluminator. The PCR products were then purified for direct DNA sequencing using the BigDye^®^ Terminator v3.1 Cycle Sequencing Kit (Applied Biosystems, Waltham, MA, USA). The sequences of the fungal isolates were identified using Basic Local Alignment Search Tool (BLAST) searches (http://www.ncbi.nlm.nih.gov/BLAST, accessed 29 October 2021). All the sequence data of the isolated soil-borne fungi were deposited in the GenBank nucleotide sequence databases. 

### 2.2. In Vitro Dual Culture Bioassays

The antagonistic potential of 35 soil-borne fungal isolates against *R. microporus*, isolate RL21) was verified in a dual culture assay with 90 mm Petri plates containing the PDA medium, referring to the method published by Fokkema [33]. Pure cultures of actively growing pathogens and potential antagonists that were ten days old, were each cut on the edge side to obtain a 7 mm diameter mycelial disc for plate seeding. *R. microporus* and antagonist plugs were positioned together on opposite sides in the same plate 1 cm away from the side of the Petri dish. To compensate for the faster growth, antagonist isolates were placed in the plate a day after the pathogen has been placed. For control samples, only a pathogen plug was placed on the plate. Inoculated plates were then sealed with Parafilm and incubated in the dark at 28 ± 2 °C. All dual cultures and control were replicated four times. On day 5 after the antagonists were inoculated, the pathogen radial growth in each plate was recorded using a caliper. The percentage inhibition of radial growth (PIRG) was calculated using Equation (1) adapted from Korsten et al. [34]:

PIRG (%) = [(r1 − r2)/r1] × 100 (1)

where

r1 = the colony radius of the pathogen in control and
r2 = the colony radius of the pathogen in treatment.

The interaction assays were scored according to Bell’s scale [26], where

R1 = 100% overgrowth;
R2 = 75% overgrowth;
R3 = 50% overgrowth;
R4 = growth inhibition at the line of contact; and
R5 = the pathogen over the growing antagonist.

### 2.3. Characterization of Trichoderma Isolates

#### 2.3.1. Antifungal Activities

##### Non-Volatile Compounds

Non-volatile substances produced by *Trichoderma* isolates were analyzed using the method published by Dennis and Webster [35] with slight modifications. The culture plates of *Trichoderma* isolates were incubated for seven days at 28 ± 2 °C for growth and metabolite production. A 7 mm diameter disc of the bioagents was then cut from an actively growing region of the culture and seeded in 100 mL of potato dextrose broth (PDB) (Oxoid Limited, Loughborough, Leics, UK). The PDB cultures were placed in a rotary shaker (Lab Line, USA) with agitation rates at 150 rpm at 28 ± 2 °C for seven days. After this time, the culture broth was strained through a sterilized Whatman filter paper No. 1 (Merck, Darmstadt, HE, Germany) and passed through a sterile membrane filter with a pore size of 0.45 µm. The filtrates were combined into a primarily prepared PDA medium which was sterilized by autoclaving and later maintained at 50 °C to avoid solidification. Three different concentrations of culture filtrate at 25%, 50%, and 75% were prepared by utilizing the PDA medium as a solvent and were then poured and solidified in Petri plates. A one-week actively grown *R. microporus* with a mycelial disc of approximately 7 mm in diameter was then transferred to the solidified agar plates containing culture filtrates of different concentrations. The inoculated agar plates were later incubated at 28 ± 2 °C. Agar plates without culture filtrates served as the control. Four replications were conducted for each treatment. The radial growth of *R. microporus* was recorded daily for the duration of one week and the PIRG value was calculated using Equation (1).

##### Volatile Compound

The effect of volatiles released from *Trichoderma* isolates in suppressing the fungal growth of plant pathogens was determined using an overlapping plate assay adapted from Boubekeur et al. [36] and Dennis and Webster [35]. A mycelial plug (7 mm in diameter) of *R. microporus* isolate RL21 was removed from the mother culture plate and inserted centrally onto the PDA Petri plate and incubated for three days. After three days, culture plates inoculated with *R. microporus* were inverted on top of the culture plates containing seven-day-old actively growing *Trichoderma* isolates. Under aseptic conditions, the Petri plates were sealed with parafilm tape and incubated in the dark at 28 ± 2 °C. For the control, the culture plate inoculated with *R. microporus* was inverted on top of the plate containing just PDA. Four replications were conducted for each treatment and the radial growth of *R. microporus* was documented daily. The inhibition rate was calculated using Equation (1).

##### Identification of Antifungal Metabolites

*Trichoderma* isolates were characterized for their secondary metabolite productions through Gas Chromatography–Mass Spectrometry (GC-MS), as described by Siddiquee et al. [37] with slight modification. For metabolite production, the flasks containing 100 mL of PDB were seeded with *Trichoderma* plugs (7 mm in diameter) and incubated at 28 ± 2 °C on a rotary shaker at 150 rpm for 21 days. The culture filtrates of *Trichoderma* isolates were collected by straining them through Whatman filter paper No. 1 (Merck, Germany) and then they passed through a sterile membrane filter with a pore size of 0.45 µm. Ethyl acetate (100 mL) with equal volume was added to the culture filtrate in a 250 mL Erlenmeyer flask, and the mixture was allowed to stand overnight to make sure the cells were dead.

The next day, the ethyl acetate phase was separated from the broth (water phase) using a separatory funnel to remove residual salts and other polar components. The separatory funnel was rinsed twice with ethyl acetate. Approximately 1–2 g of sodium sulfate salt was added to the collected ethyl acetate phase to remove excess water. It was then separated from the ethyl acetate phase using Whatman filter paper No. 1 (Merck, Germany). The ethyl acetate phase was further evaporated using a rotary evaporator at 40 °C in a 250 mL round bottom flask. The crude extract was then diluted with 100 mL of 90% methanol and 100 mL of n-hexane. n-hexane was added to remove fatty acids and other non-polar components. The two different layers that formed were then separated using a separatory funnel. The methanol phase formed at the bottom was collected and further evaporated in a 250 mL round bottom flask at 40 °C using a rotary evaporator. Extracted compounds formed in the round bottom flasks were then diluted with 10 mL of high-performance liquid chromatography (HPLC) grade methanol. The extracted compounds were stored in a −20 °C freezer until further use. 

Hydrocarbons and other volatile compounds were separated using the TSQ Quantum Instrument Method (Thermo Scientific, Waltham, MA, USA). GC-MS analysis was performed at an ionization energy of 70 eV. A TG-5MS column (Thermo Scientific, USA) (30 m × 0.25 mm ID, 0.25 µm film thickness) was used to separate volatile compounds produced by *Trichoderma* isolates at a temperature of 260 °C. The oven conditions were 1 min for equilibration time and 40 °C for 2 min, and the program was further ramped to 200°C at a rate of 10 °C/min and 25 °C/min to 260 °C for 25 min, with a total run time of 45.40 min. Identified volatile compounds produced by *Trichoderma* isolates were compared with the mass spectra from a reference library (NIST-17 Mass Spectra Library, National Institute of Standards and Technology).

#### 2.3.2. Production of Extracellular Hydrolytic Enzymes 

##### Chitinase

For the quantitative assessment of chitinase activity, the crude extracts of *Trichoderma* isolates were prepared by culturing the antagonists in 100 mL of *Trichoderma* Liquid Enzyme (TLE), which comprised KH_2_PO_4_ (0.2 g/L), (NH_4_)_2_SO_4_ (0.14 g/L), bactopeptone (0.1 g/L), urea (0.03 g/L), MgSO_4_·7H_2_O (0.03 g/L), CaCl_2_·6H_2_O (0.03 g/L), and 1 mL of a 0.01% trace elements solution (Fe^2+^, Mn^2+^, Zn^2+^, and Co^2+^). A total of 1 g of colloidal chitin was added to the TLE as a source of carbon. The fungal cultures were incubated for 72 h at 28 ± 2 °C with an agitation rate of 150 rpm. All microbial cultivation was performed in triplicate for each of the isolates. At the end of the incubation, the culture supernatants and mycelia were separated by filtration through Whatman filter paper No. 1 (Merck, Germany). The chitinase activities of *Trichoderma* isolates were determined using 1% (*w*/*v*) colloidal chitin dissolved in a 0.05 M sodium acetate buffer (pH 5.2)). The reaction mixtures which contained 50 µL of enzyme solution (culture supernatants) and 100 µL of colloidal chitin dissolved in the buffer were used to run the enzyme detection. The mixtures were then incubated for 15 min at 37 ± 2 °C. At the end of the incubation, 0.5 mL of 3,5-dinitrosalicylic acid (DNS reagent) was added and heated at 95 °C for 10 min to stop the enzyme–substrate reaction. N-acetylglucosamine was used for the preparation of the standard curves. The series of N-acetylglucosamine solutions were added with 1.0 mL of the DNS reagent and placed in a boiling water bath for 5 min. The absorbance values of the solutions were read at a 540 nm wavelength using Varian Cary 50 UV-Vis spectrophotometer (Varian, Mulgrave, Victoria, Australia). The concentration of reducing sugar for each sample was calculated using the standard curves regression equation and the results are expressed in mg/mL. 

The qualitative analysis of chitinase production by *Trichoderma* isolates was conducted based on the protocol published by Murthy and Bleakley [38], with slight modifications. Purified chitin powder (R & M chemicals, Essex, MA, UK) was used to prepare colloidal chitin and incorporated into the chitinase agar medium as a sole carbon source. About 5 g of chitin powder was slowly added into 60 mL of concentrated HCl (Fisher Scientific, Loughborough, Leics, UK) and stirred vigorously at room temperature for 1 h. The solution was then filtered using glass wool in a glass funnel. A total of 200 mL of 50% ethanol was added to the filtrate with vigorous stirring to precipitate the filtrate. The precipitate was then collected by passing through Whatman filter paper No. 1 (Merck, Germany). Colloidal chitin was collected from the filter paper and stored at 4 °C in the dark until further used. The chitinase detection medium, which consisted of a mixture of colloidal chitin (4.5 g/L), MgSO_4_·7H2O (0.3 g/L), (NH_4_)_2_SO_4_ (3.0 g/L), KH_2_PO_4_ (2.0 g/L), citric acid monohydrate (1.0 g/L), Bacto agar (15 g/L), bromocresol purple (0.15 g/L), and Tween-80 (200 μL/L) was adjusted to pH 4.7 and then autoclaved at 121 °C for 15 min. Seven-day-old culture plugs (7 mm) were seeded in the center of the respective medium and incubated at 28 ± 2 °C, with four replications per isolate. The Color intensity and diameter of the purple-colored zone (chitinolytic activity) around the colony of *Trichoderma* isolates were observed. The enzyme activity was ranked using a scale from 0, the isolate showing no activity to 4, the isolate showing the highest activity.

##### Cellulase

For the quantitative assessment of cellulase activity, the crude extracts of *Trichoderma* isolates were prepared by culturing the antagonists in a liquid basal medium containing 10 g of glucose, 5 g of yeast extract, 0.6 g of KH_2_PO_4_, 0.5 g of MgSO_4_·7H_2_O, 0.4 g of K_2_HPO_4_, 0.25 g of CuSO_4_, 0.05 g of FeSO_4_·7H_2_O, 0.05 g MnSO_4_, and 0.001 g of ZnSO_4_ prepared in 1 L of distilled water, as described by Tellez-Tellez et al. [39]. The fungal cultures were incubated for 72 h at 28 ± 2 °C with an agitation rate of 150 rpm. All microbial cultivation was performed in triplicate for each of the isolates. At the end of the incubation, the culture supernatants and mycelia were separated by filtration through Whatman filter paper No. 1 (Merck, Germany). The cellulase activities of *Trichoderma* isolates were determined using 1% (*w*/*v*) carboxymethyl cellulose (CMC) dissolved in a 0.05 M citrate buffer (pH 4.0). The reaction mixtures, which contained 50 µL of enzyme solution (culture supernatants) and 100 µL of CMC dissolved in a citrate buffer, were used to conduct the cellulase activities. The mixtures were then incubated for 15 min at 37 ± 2 °C. At the end of the incubation, 0.5 mL of 3,5-dinitrosalicylic acid (DNS reagent) was added and heated at 95 °C for 10 min to stop the enzyme–substrate reaction. Glucose was used for the preparation of the standard curves. The series of glucose solutions were added with 1.0 mL of the DNS reagent and placed in a boiling water bath for 5 min. The absorbance values of the solutions were read at a 540 nm wavelength using Varian Cary 50 UV-Vis spectrophotometer (Varian, Mulgrave, Victoria, Australia). The concentration of reducing sugar for each sample was calculated using the standard curves regression equation and the results are expressed in mg/mL. 

The qualitative analysis of cellulase production by *Trichoderma* isolates was determined by referring to the method described by Zehra et al. [40]. The (CMC) agar was prepared in 1 L of distilled water consisting of 16 g of the Bacto agar, 0.5 g of CMC, 0.5 g of bactopeptone, 0.2 g of Congo red, and 0.1 g of yeast extract. The antagonists (7 mm plugs) were inoculated on the respective media and incubated at 28 ± 2 °C for 4–7 days with four replications each. CMC agar plates were observed for the yellow, opaque, or discolored zone (cellulase activity) around the colony. The enzyme activity was ranked using the score as described in the chitinase assay. 

##### β-1,3-glucanase

For the quantitative assessment of β-1,3-glucanase activity, the crude extracts of *Trichoderma* isolates were prepared by culturing the antagonists in 100 mL of *Trichoderma* Liquid Enzyme (TLE), which comprised KH_2_PO_4_ (0.2 g/L), (NH_4_)_2_SO_4_ (0.14 g/L), bactopeptone (0.1 g/L), urea (0.03 g/L), MgSO_4_·7H_2_O (0.03 g/L), CaCl_2_·6H_2_O (0.03 g/L), and 1 mL of 0.01% trace element solution (Fe^2+^, Mn^2+^, Zn^2+^, and Co^2+^). A total of 0.3 g of glucose and 0.5 g of colloidal chitin were added to the TLE as a source of carbon. The fungal cultures were incubated for 72 h at 28 ± 2 °C with an agitation rate of 150 rpm. All microbial cultivation was performed in triplicate for each of the isolates. At the end of the incubation, the culture supernatants and mycelia were separated by filtration through Whatman filter paper No. 1 (Merck, Germany). The β-1,3-glucanase activities of *Trichoderma* isolates were determined using 0.75% (*w*/*v*) laminarin dissolved in a 0.05 M sodium acetate buffer (pH 5.2). The reaction mixtures, which contained 50 µL enzyme solution (culture supernatants) and 100 µL substrates dissolved in respective buffers, were used to run the enzyme detection. The mixtures were then incubated for 15 min at 37 ± 2 °C. At the end of the incubation, 0.5 mL of 3,5-dinitrosalicylic acid (the DNS reagent) was added and heated at 95 °C for 10 min to stop the enzyme–substrate reaction. Glucose was used for the preparation of the standard curves. The series of glucose solutions were added with 1.0 mL of the DNS reagent and placed in a boiling water bath for 5 min. The absorbance values of the solutions were read at a 540 nm wavelength using a spectrophotometer (Varian Cary 50 Conc, Australia). The concentration of reducing sugar for each sample was calculated using the standard curves regression equation and the results are expressed in mg/mL. 

#### 2.3.3. Plant Growth Promotion Activity

##### Indole Acetic Acid Production

Indole acetic acid (IAA) produced by *Trichoderma* isolates was quantified by following the protocol as described by Noori and Saud [41]. *Trichoderma* isolates were grown in sterile PDB with 5 mL of 0.2% (*w*/*v*) L-tryptophan, incubated at 28 ± 2 °C, and allowed to grow for 72 h. A conical flask without a fungal plug served as the control. Culture supernatants were filtered with Whatman filter paper No. 1 (Merck, Germany) after the optimum period (72 h). A total of 1 mL of filtrate was mixed with 2 mL of Salkowski reagent (35% perchloric acid, 1 mL 0.5 M FeCl_3_). After 20 min incubation, the color density formed was read using a UV spectrophotometer at a 540 nm absorbance. The appearance of red or pink color indicated IAA production. To determine the amount of IAA produced from *Trichoderma* isolates, a series of IAA dilutions generated at 0, 5, 10, 20, 50, and 100 µg/mL was prepared to plot a standard curve. The presence of IAA in the culture filtrate was compared to the standard graph and expressed as µg/mL.

##### Phosphate Solubilization Test

The ability of *Trichoderma* isolates in solubilizing phosphate was analyzed using the Pikovskaya (PVK) agar medium of the following components: Ca_3_(PO_4_)_2_ (5 g/L), (NH_4_)_2_SO_4_ (0.5 g/L), KCl (0.2 g/L), MgSO_4_·7H_2_O (0.1 g/L), MnSO_4_ (0.0001 g/L), FeSO_4_·7H_2_O (0.0001 g/L), yeast extract (0.5 g/L), dextrose (10 g/L), and the Bacto agar (15 g/L) [18]. The agar medium was then autoclaved at 121 °C for 15 min. *Trichoderma* isolates with a 7 mm diameter disc were transferred in the center of the PVK agar medium and incubated for 3–5 days at 28 ± 2 °C, with four replications per isolate. The Halo zone (solubilizing zone) around the fungal plugs indicated positive solubilization of phosphate [41].

##### Siderophores Production 

The chrome azurol S (CAS) assay described by Schwyn and Neilands was used to detect the production of siderophores. The CAS agar was prepared by mixing 900 mL of King’s B medium and 100 mL of a CAS-HDTMA solution. King’s B medium was prepared using 20 g of the Bacto agar, 20 g protease peptone, 1.5 g MgSO_4_·7H_2_O, and 1.5 g K_2_HPO_4_, in 1 L of distilled water. The CAS-HDTMA solution was prepared using 0.062 g of CAS in 50 mL dH_2_O, 0.072 g hexa decyl tri methyl-ammonium bromide (HDTMA) in 40 mL dH_2_O, and 10 mL of iron (III) solution (1 mM FeCl_3_ in 10 mM HCl). The CAS agar medium was sterilized by autoclaving at 121 °C for 15 min. Fungal plugs (7 mm in diameter) were plated on the CAS agar medium and incubated for 4–7 days at 28 ± 2 °C. Four replications were conducted per isolate. The amount of siderophore production activity was evaluated based on the orange zone developed around the fungal colony, according to Cherkupally et al. [42].

#### 2.3.4. Biochemical Properties Index

A scoring system was assigned based on the mode of action which includes mycoparasitism, antifungal activities, production of extracellular hydrolytic enzymes, and plant growth-promoting activities associated with *Trichoderma* isolates against *R. microporus*. A scale order was used to objectively rank the activity of each *Trichoderma* isolate ranging from 0 to 4, with 4 indicating the isolate showing the highest activity, 3 showing the isolate with the second highest activity, 2 indicating the isolate at third place, 1 indicating the isolate showing the lowest activity, and 0 indicating the isolate showing no activity. Isolates with identical values were assigned an identical rank score. If a pair of isolates tie for the highest score, both isolates were assigned under scale 4 and the next isolate activity value will not be ranked under scale 3 but instead ranked under scale 2. The scoring results were used to select the potential *Trichoderma* isolates and further explored as BCAs for the evaluation of their efficiency against *R. microporus* under a nursery experimental plot.

### 2.4. Nursery Experiments

#### 2.4.1. Biocontrol and Pathogen Inoculum Preparation

*Trichoderma asperellum* ST011 and *T. spirale* HT009 isolates were selected as potential BCAs to evaluate their capability against *R. microporus* in vivo. The *Trichoderma* isolates were grown on a PDA medium at 28 ± 2 °C for one week. Sterilized distilled water was added onto the PDA plate of *Trichoderma asperellum* ST011 and *T. spirale* HT009, and the fungal mass was scraped from the culture plate using a fine sterilized spatula to prepare a stock solution for serial dilution. A serial dilution was performed to quantify the total number of spores on a single plate. The colony-forming units (cfu) for each of the dilutions were determined by spore counting using a hemocytometer under the microscope. At the time of application, the spore concentration of each BCAs was adjusted to 10^8^ cfu/mL using sterilized distilled water.

Wood for inoculation was obtained from a freshly cut rubber tree in UPM. The logs were then cut into smaller blocks with dimensions of 6 cm × 6 cm × 6 cm. Wood blocks were first washed with tap water and autoclaved twice at 121 °C, 15 psi for 30 min. A melted (liquefied by heat) malt extract agar (MEA) (Oxoid, UK) of 60 mL was poured into a high-performance plastic (HPP) bag (7″ × 10″ dimension) containing a rubber block. A MEA was added as a supplementary nutrient for the growth of *R. microporus*. Cotton plugs were used to plug the open end of the bags to allow the passage of air, but prevent any contamination of the blocks. The bags containing wood blocks and melted MEA were autoclaved at 121 °C and 15 psi for 20 min. 

During the cooling process, the HPP bags were rotated frequently to ensure the wood blocks were well coated with the melted agar before the agar solidified. Each of the rubber wood blocks coated with MEA was inoculated with a 1 cm^2^ fungal plug taken from a plate of one-week-old *R. microporus* culture. The inoculated blocks were incubated in dark conditions for one month at 28 ± 2 °C until the blocks were fully colonized by *R. microporus* mycelium. 

#### 2.4.2. Rubber Seedlings Inoculation

RRIM600 was selected as the host plant due to its high susceptibility to *R. microporus* [29] which favored WRD development. Four-month-old *H. brasiliensis*, clone RRIM600 seedlings, were bought from Sendayan Nursery, Seremban. The seedlings were maintained in nursery plots of the Faculty of Forestry, Universiti Putra Malaysia (UPM) for two months prior to the nursery assessment. The rubber seedlings were watered daily and fertilized once with NPK (15:15:15). Neem oil was applied to the plant surface using a 5 L garden pressure sprayer to eliminate any pest infestation. 

In vivo study was carried out at the nursery of the Faculty of Forestry, UPM. The study was carried out using randomized complete block design with four blocks and six treatments (Table 1) for six replicates randomized within each block (*n* = 4). The six-month-old rubber seedlings of the clone RRIM600 were maintained in polybags (10-inch × 12-inch) which contained a 7 kg/polybag mixture of soil, sand, and compost at a ratio of 8:8:2 as a growth substrate. The planting media mixture for the seedlings in all treatments was autoclaved twice at 121 °C for 1 h. For treatment T3 (*T. asperellum* ST011), treatment T4 (*T. spirale* HT009), and treatment T5 (*T. asperellum* + *T. spirale*), biocontrol suspensions of 100 mL per polybag were poured evenly into the sterilized planting media once a week for a period of one month before *R. microporus* inoculation. For chemical treatment (T6), the fungicide Kentil propiconazole 250 EC (Kenso Corporation, Petaling Jaya, Selangor, Malaysia) was diluted with distilled water at a ratio of 1:100 and 100 mL of the dilution was poured evenly into the soil once a week for a period of one month before *R. microporus* inoculation. 

The inoculations of *R. microporus* were started 7 days after the last biocontrol suspension or fungicide dilution was added to the planting media. The inoculation was performed by placing *R. microporus* inoculum (rubber blocks colonized by *R. microporus* mycelium) at the bottom of the polybag, which was in direct contact with the roots of the rubber seedlings. The polybags were then filled up with the planting media treated with biocontrol suspension/fungicide for treatments 3–6. Planting medium inoculated with the pathogen but without biocontrol suspension/fungicide served as positive control and seedlings without *R. microporus* inoculum served as a negative control. The seedlings were then placed on benches in the nursery and were watered daily.

#### 2.4.3. Disease Severity Assessment

The disease assessment was adapted from the method published by Wattanasilakorn et al. [43]. The plants challenged with *R. microporus* were observed for the growth of fungal rhizomorphs at the root and collar region of the plants. The symptoms developed on the roots (rotted) and the leaves (yellowing) were recorded. Observations were conducted at one-month intervals for six months after one month of the preliminary incubation period. The time to establish the disease symptoms and signs was also recorded. Four seedlings from each treatment, with one plant per block, were harvested at monthly intervals for disease assessment. For each isolate, the disease severity index (DSI) was determined using Equation (2) based on the infection scores, which include justifications of above- and below-ground symptoms, as shown in Table 2.

Disease severity index (DSI) % = (S_0_ × X_S0_) + (S_1_ × X_S1_) + (S_2_ × X_S2_) + … (S_H_ × X_SH_) × 100 (2)

X_sum_ × S_H_

where

S_0_, S_1_, and S_2_, …, S_H_ are the disease severity scores (0 to 4 for above-ground symptoms and 0 to 5 for below-ground symptoms),
X_S1_, X_S2_, and X_S3_, …, X_SH_ are number of plants that are classified under the specific disease severity scoring,
X_sum_ is the total of plants, and
S_H_ is the the highest scoring of disease severity classification: 4 for above-ground symptoms or 5 for below-ground symptoms.

The classification scheme used in the disease severity index (DSI) was categorized with four disease severity scorings for above-ground symptoms and five disease severity scorings for below-ground symptoms (Table 2). The efficacy of biocontrol or chemical application was then calculated based on the average DSI obtained from each of the treatments using Equation (3), as described by Gafni et al. [44]:

Efficacy (%) = (*s*1 − *s*2)/*s*1 × 100 (3)

where

s1 = Disease severity index in the positive control and
*s*2 = Disease severity index in the biocontrol or chemical treatment.

#### 2.4.4. Transmission Electron Microscopy (TEM) Observation 

The tissue processing method for the TEM was adapted from Kim and Kremer [39] with slight modifications. Small root fragments of about 2 mm long x 1 mm thickness were excised from the harvested plant. Healthy and infected root tissues were fixed in 2.5% glutaraldehyde at 4 °C overnight. The root tissues were again fixed in 2.5% glutaraldehyde at 4 °C for 2 h. The samples were then washed with a 0.1 M sodium cacodylate buffer for 10 min and the washing step was repeated three times. This was followed by a post-fixed in 1% osmium tetroxide at 4 °C for 2 h. The samples were subsequently washed again with a 0.1 M sodium cacodylate buffer for 10 min and repeated three times. The dehydration process was performed in a crescent series of acetone solutions (35, 50, 75, 95, and 100%) for 10 min each up to 95% concentration, followed by 15 min with 100% concentration and repeated three times. The preparation process was continued by infiltration of the samples with an acetone and resin mixture. The resin was made up of 10 mL of agar 100 resin, 6 mL of dodecenylsuccinic anhydride, 5.5 mL of methyl nadic anhydride, and 0.5 mL benzyldimethylamine. The samples were immersed in the acetone and resin mixture at a ratio of 1:1 for 4 h, followed by 1:3 overnight, and immersed overnight with resin. The samples were then kept in beam capsules and filled with resin. Next, polymerization was performed in the oven at 60 °C for 48 h. Thin sections (1 µm) were sliced using an ultramicrotome with a diamond knife. The sections were stained with uranyl acetate for 15 min and washed with double-distilled water (ddH_2_O), followed by lead citrate for 10 min and washed with ddH_2_O. The sections were examined with a JEOL JEM2100F Field Emission Transmission electron microscope (JOEL, Japan). The root vascular system of the rubber seedlings and white root rot pathogen colonization patterns were observed by TEM.

### 2.5. Statistical Analysis

Statistical analyses were conducted using the statistical package SPSS for Windows, version 23.0 (SPSS, Chicago, IL, USA), which was used to evaluate the quantitative data of the PIRG values for a dual culture assay, non-volatile, and volatile metabolites; and the biochemical assays which include chitinase, cellulase, β-1, 3-glucanase enzymes, and IAA productions for analysis of variance (ANOVA) at a 95% confident level (*p* ≤ 0.05). Results were analyzed using one-way ANOVA, followed by Tukey’s test as a post hoc test. The Tukey–Kramer multiple comparisons test was applied to analyze the differences between the treatment effects when significance was observed. The effects were considered to be not statistically significant when the *p*-value was higher than 0.05 at a 95% confidence level.

## 3. Results and Discussion

### 3.1. Identification and Selection of Potential Antagonistic Fungi through Dual Culture Assays

A total of 35 fungal isolates were successfully established from the soil samples obtained from the rhizosphere of rubber trees located at RRIM, Sungai Buloh. The sequence data of the fungal species associated with the soil sampled in this study was deposited in the GenBank nucleotide sequence databases (Table 3).

**Figure 1:**
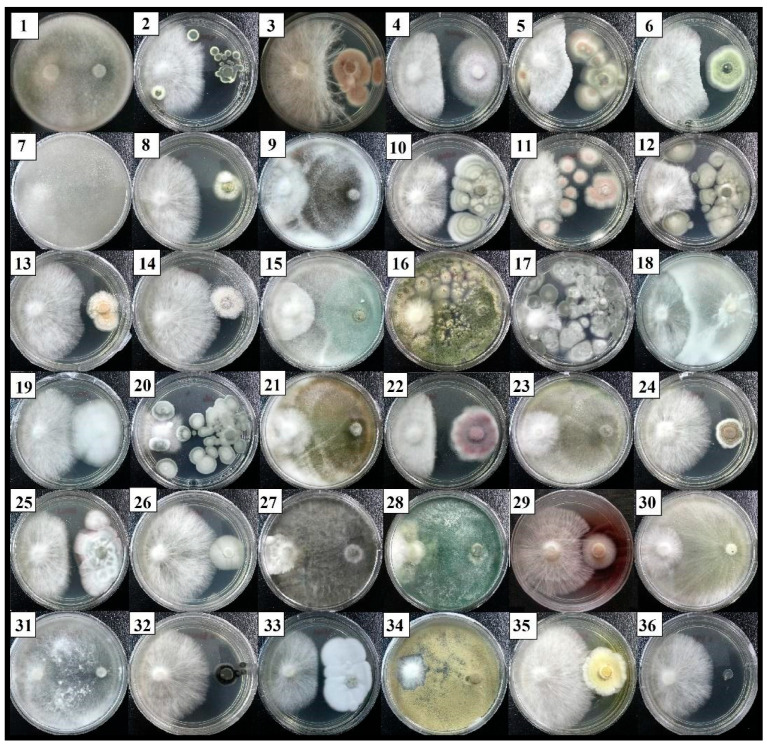
Interaction between *Rigidoporus microporus* (**left**) and 35 isolates of soil-borne fungi (**right**) after 5 days of dual culture in Petri dishes with the potato dextrose agar. See Table 4 for the fungal–number association.

Figure 1 shows the direct competitive interaction by a dual culture assay of 35 fungal isolates against the growth of an *R. microporus* isolate RL21. The isolates HT007 and ST011, which were identified as *Cunninghamella bainieri* and *T. asperellum,* revealed an aggressive inhibitory result on the hyphal growth of *R. microporus* after five days of incubation, with significantly, the highest PIRG value recorded by *Cunninghamella bainieri* at 93.18% (Table 4). This was followed by *T. asperellum* (ST011) and *L. theobromae* (ST010). Other isolates that performed strong antagonism with PIRG values of more than 75% against *R. microporus* were identified as *T. koningiopsis* (isolates HT001 and ST014) and *T. spirale* (HT009).

The first apparent physical contact between the above isolates and the pathogen *R. microporus* occurred within the first 2–3 days, followed by growth inhibition. Figure 1 shows that the isolates HT007, ST011, HT001, ST014, HT009, and ST010 have fully occupied the PDA medium and overgrowth the pathogen on the 5th day after inoculation (DAI). This caused a space limitation for *R. microporus,* preventing any radial expansion on the culture plate. The present study showed that there were four *Trichoderma* spp., namely *T. asperellum, T. koningiopsis*, *T. reesei,* and *T. spirale,* together with *C. bainieri*, *Lasiodiplodia theobromae*, *Aspergillus nomius*, *Byssochlamys spectabilis,* and *Penicillium* sp. categorized under R1 and R2 (Table 4). With these scores (R1 and R2), the mentioned isolates were considered antagonistic to the pathogen, with a substantial PIRG result achieved by the isolates HT007 and ST011. 

Moderate antifungal activities were found in the other ten isolates, which include *T. koningiopsis* (HT015), *A. nomius* (HT016), *Penicillium* sp. (HT017 and ST003), *B. spectabilis* (HT018 and ST017), *T. spirale* (ST004 and ST006), *Fusarium oxysporum* (ST005), and *T. reesei* (ST013). These isolates inhibited the growth of *R. microporus* by 52.18–70.35% within five DAI. Among these, *F. oxysporum* (ST005) created the most distinct growth inhibition zone at the line of contact with the pathogen. However, some changes were observed in the physical interactions between isolate ST005 with *R. microporus* after a growing period of 15 days. The pathogen was not just capable to circumvent parasitism but also formed mycelial strands to grow over the mycelia of isolate ST005. 

The remaining thirteen isolates belonging to the genus *Penicillium*, *Purpureocillium,* and *Talaromyces* revealed weak inhibition, with PIRG results of less than 50%. No inhibitory effect against *R. microporus* was recorded by *Clonostachys* sp. (HT013 and ST018), *Scedosporium boydii* (HT014), *Xepicula leucotricha* (ST007), *Trichosporiella* sp. (ST009), and *Wiesneriomyces laurinus* (ST015). This could be due to their slower growth rate.

Despite the promising results demonstrated by *C. bainieri* and *L. theobromae* for their antagonism against *R. microporus*, both isolates were not taken into consideration as potential biocontrol agents. This is due to the genus of the species *Cunninghamella* being reported to cause infections, especially to the immunocompromised hosts [45], and *L. theobromae* to cause diseases, such as die-back, blight, and root rot to different crops in the tropical and subtropical regions, which includes guava, coconut, papaya, and grapevine [46].

### 3.2. Characterization of Antifungal Activity of selected Trichoderma Isolates

#### 3.2.1. Non-Volatile Compounds

**Figure 2:**
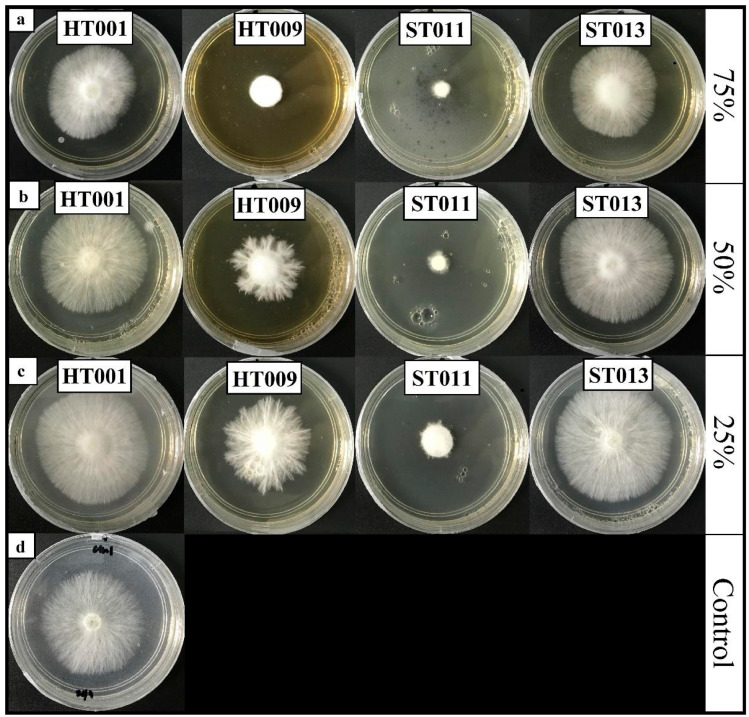
Effects of culture filtrate of *Trichoderma koningiopsis* (HT001), *Trichoderma spirale* (HT009), *Trichoderma asperellum* (ST011), and *Trichoderma reesei* (ST013) assayed at concentrations of (**a**) 75, (**b**) 50, and (**c**) 25% in the potato dextrose agar against *Rigidoporus microporus*. Pictures referred to 5 days after inoculation. (**d**) Control plate.

The results of the study revealed a significant interaction (*p* ≤ 0.05) between the *Trichoderma* species and the concentration of non-volatile filtrate (*Trichoderma* isolates x the concentration of culture filtrate) on the growth inhibition of *R. microporus* (Figure 2). Isolate *T. asperellum* extracts with 75% and 50% concentrations of culture filtrates exhibited the highest inhibition activity against *R. microporus* after five DAI, with the inhibition rate recorded at 78.80% and 77.41%, respectively (Table 5). The non-volatile filtrate of *T. koningiopsis* HT001 and *T. reesei* ST013 was recorded to have the least effect against *R. microporus,* with minimal inhibition rates at 10.20% and 5.90%, respectively, even with a 75% concentration. No inhibition activity was observed against the pathogen in the plates incorporated, with 50% and 25% of the culture filtrate produced by *T. koningiopsis* HT001 and *T. reesei* ST013.

Figure 2 shows the effect of non-volatile filtrates produced by *Trichoderma* spp. on the morphological characteristic of *R. microporus*. Distorted shapes on the growth patterns and sparse mycelial growth were clearly seen, especially in the non-volatile filtrate plates of *T. spirale* HT009 and *T. asperellum* ST011, as compared to the control at five DAI (Figure 2d). The study documented by Dixit et al. [47] demonstrated the abnormal morphological characters of the pathogenic fungi, i.e., the reduced number of sclerotia and sparse mycelial growth was due to the presence of an inhibiting substance produced by the *Trichoderma* spp. The lack of inhibitory effects against *R. microporus* with a non-volatile compound in *T. koningiopsis* HT001 and *T. reesei* ST013 isolates indicated that their antagonism activities were performed using other mechanisms rather than in non-volatile compound production. 

#### 3.2.2. Volatile Compounds

Volatile compounds produced by *Trichoderma* isolates induced a slight inhibition of the pathogen growth (Table 6). The inhibition of *R. microporus* demonstrated by *Trichoderma* isolates was moderate to weak, at which *T. asperellum* ST011 performed at 55.77%, followed by *T. spirale* HT009 (37.77%), *T. koningiopsis* HT001 (33.42%), and *T. reesei* ST013 (26.76%), respectively (Table 6). 

Volatile secondary metabolites have been reported to play a major role in the mycoparasitism of Trichoderma spp. [48]. The highest number of compounds was perceived from T. asperellum ST011 (40 compounds) followed by 30 peaks from each of the isolates T. koningiopsis HT001, T. spirale HT009, and T. reesei ST013. The main components (peak area > 1%) with a match quality ≥ 60% in the NIST-17 library search were listed in Table 7.

Compound classes such as alcohols, phenols, acids, esters, and amides were detected in the volatile compounds from the culture samples. The most abundant compounds identified were Dodecanoic acid,1,2,3-propanetriyl ester in the *T. koningiopsis* HT001, (5á)Pregnane-3,20á-diol,14à,18à-[4-methyl-3-oxo-(1-oxa-4-azabutane-1,4-diyl)]-, diacetate in *T. spirale* HT009, 13-Docosenamide,(Z)-, and 1-Dodecanoyl-3-myristoyl glycerol were recorded in *T. asperellum* ST011. The compound 13-Docosenamide,(Z)- was detected in the extracts of all *Trichoderma* isolates. The benzenepropanoic acid,3,5-bis(1,1-dimethylethyl)-4-hydroxy- methyl ester was identified from the extracts of the three isolates; i.e., *T. koningiopsis* HT001, *T. spirale* HT009, and *T. reesei* ST013.

Undoubtedly, the chemical profiles varied among different isolates. The common compound 13-Docosenamide,(Z)- identified in the present study was reported to have an antimicrobial function when it was extracted from the seeds of *Mucuna pruriens* Linn [46] and Zhang et al. [49] also stated that 13-Docosenamide,(Z)- was one of the key bioactive compounds produced by *T. longibrachiatum,* with its antifungal activity recorded at 7.69%. 

2,4-Di-tert-butylphenol detected in *T. koningiopsis* HT001, *T. asperellum* ST011, and *T. reesei* ST013 was reported to possess antifungal and antioxidant activities, at which it was secreted by *Lactococcus* sp. [48]. Four isolates of *T. asperellum* have been reported by Srinivasa et al. [50] to produce 2,4-Di-tert-butylphenol. *Flavobacterium johnsoniae* was also reported by Sang and Kim [51] to produce 2,4-Di-tert-butylphenol that exhibits inhibition against *Phytophthora capsici* at various concentrations.

The compound 4-hydroxy-benzeneethanol identified in the crude culture of *T. asperellum* ST011 and *T. reesei* ST013 was previously reported to decrease the root-knot nematode egg hatchability to less than 10% with a 9.6 mM or higher concentration of the active compound extracted from Welsh onion root exudates [52].

The Octadecenamide,(Z)- compound detected in the methanolic extracts of isolates *T. spirale* HT009 and *T. asperellum* ST011 has been reported by Haider et al. [53] to have anti-inflammatory and antibacterial properties extracted from leaves of the Southern maidenhair fern, *Adiantum capillus-veneris*. The compound named Estra-1,3,5(10)-trien-17αol that was only found in the crude isolate *T. asperellum* ST011 has the activity of antifungal, antibacterial, anti-inflammatory, and antioxidant [54]. 4-hydroxy-benzeneethanol, Octadecenamide,(Z)-, and Estra-1,3,5(10)-trien-17αol were never mentioned in any previous works to be discovered from *Trichoderma* species.

#### 3.2.3. Production of Extracellular Hydrolytic Enzymes

**Figure 3:**
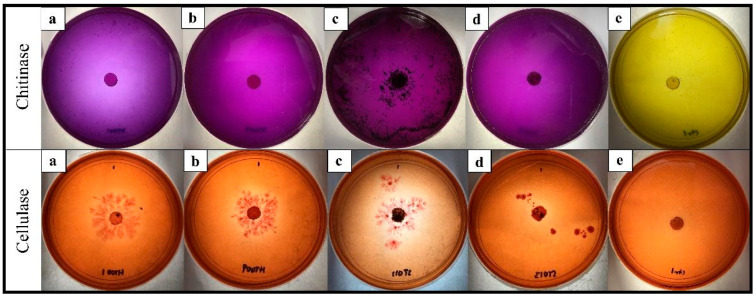
Colorimetric detection of chitinase and cellulase produced by *Trichoderma* isolates. (**a**) *T. koningiopsis* HT001, (**b**) *T. spirale* HT009, (**c**) *T. asperellum* ST011, (**d**) *T. reesei* ST013, and (**e**) the control plate on the colloidal chitin (top row) and the carboxymethyl cellulose (bottom row) agar medium. The purple-colored zone on the colloidal chitin agar plate around the colony indicates chitinase production. The formation of a yellow and opaque zone around the colony growing on the carboxymethyl cellulose agar indicates cellulase activity.

*T. asperellum* ST011 had the highest activity of all the enzymes tested (Table 8). The result of the chitinase plate assay from *T. asperellum* ST011 demonstrated a rapid and the highest response, with quantitative results at 0.04 µmol/min. The fastest reaction of color changed and the darkest purplish zone formation by *T. asperellum* ST011 was evident (Figure 3c). The other three isolates exhibited similar results for chitinase activity (0.02 µmol/min) after 72 h of incubation at 28 °C. In general, all the *Trichoderma* isolates fully changed the chitinase detection agar to purple color at five DAI.

*T. asperellum* ST011 was significantly the highest in cellulase activity (0.26 µmol/min), followed by *T. spirale* HT009 (0.18 µmol/min), *T. koningiopsis* HT001 (0.11 µmol/min), and *T. reesei* ST013 (0.04 µmol/min) (Table 8). This corresponded with the results obtained from the cellulase plate assay, at which *T. asperellum* ST011 showed a distinct yellow-opaque hydrolyzing zone on the CMC plate (Figure 3c). From the quantitative results of glucanase presented in Table 8, the highest activity was exhibited by *T. asperellum* ST011 (0.06 µmol/min), followed by *T. koningiopsis* HT001 (0.03 µmol/min), and *T. reesei* ST013 (0.02 µmol/min). No glucanase activity was shown by *T. spirale* HT009.

Chitinase and glucanase enzymes secreted by *Trichoderma* species are known to act synergistically in degrading the cell wall of the fungi pathogens so as to obtain nutrients for their own growth [55]. The present study shows that some of the *Trichoderma* isolates presented higher ability in hydrolyzing chitin, cellulose, and glucan with isolate *T. asperellum* ST011 achieving the highest score for all the enzyme activities tested. As the main component of the fungal cell wall is made up of chitin, glucan, and proteins [56], *Trichoderma* isolates that possessed higher enzyme activities have a higher capability to hydrolyze these elements, which is one of the key mechanisms in antagonistic activity for the isolate against the plant pathogenic fungi. Hence, chitinase, glucanase, and cellulase are essential in the hyperparasitic system.

#### 3.2.4. Plant Growth Promotion Activity

Significant differences were observed in the mean values of IAA production between *T. asperellum* ST011 and *T. reesei* ST013. The highest amount of IAA was produced by *T. asperellum* ST011 (1.68 μg/mL) followed by *T. reesei* ST013 (0.83 μg/mL), and was absent in *T. koningiopsis* HT001 and *T. spirale* HT009 (Table 9). IAA is essential for plant growth development [57] and enhances the fitness of plant–microbe interactions by increasing the amount of lateral and adventitious roots. This facilitates nutrient absorption and promotes root exudation, which in turn provides more opportunities for root–microbe interaction [58]. In the present study, both *T. asperellum* and *T. reesei* showed positive IAA production. A similar finding was reported by Muniroh et al. [59], stating that *T. asperellum* was able to produce IAA in lower amounts. Brummell and Hall [60] claimed that even small amounts of IAA could directly enhance plant growth by stimulating cell division or elongation.

**Figure 4:**
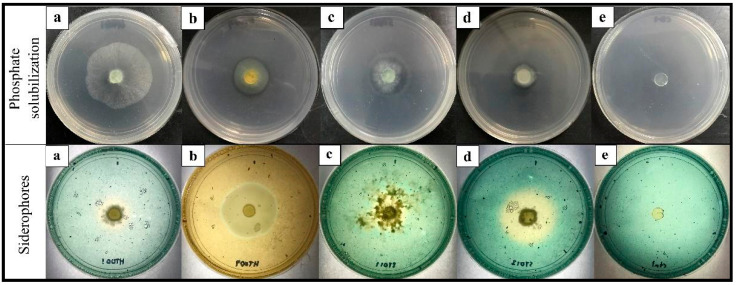
Colorimetric detection of phosphate solubilization and siderophores production by *Trichoderma* isolates. (**a**) *T. koningiopsis* HT001, (**b**) *T. spirale* HT009, (**c**) *T. asperellum* ST011, (**d**) *T. reesei* ST013, and (**e**) the control plate on the Pikovskaya (top row) and the chrome azurol S (bottom row) agar medium. The halo zone on the Pikovskaya agar plate around the colony indicates phosphate solubilization. The formation of an orange zone around the colony growing on the chrome azurol S agar indicates siderophores production.

The *Trichoderma* isolates were verified on phosphate solubilizing using a PVK agar plate. The ability to solubilize phosphate was positively exhibited by *T. spirale* HT009, *T. asperellum* ST011, and *T. reesei* ST013 to different extents (Figure 4). Phosphate solubilization was not detected from *T. koningiopsis* HT001. Phosphorus (P) is one of the important plant nutrients that has a significant impact on plant growth. In the present study, *T. koningiopsis* HT001 was the only isolate that was not able to solubilize the inorganic phosphate. The key process responsible for the release of insoluble phosphate was the generation of organic acids by the phosphate-solubilizing microbes. Phosphate-solubilizing microbes, such as fungi and bacteria, play an important role in transforming insoluble P to soluble P forms that can easily be assimilated by plants [59]. Although the nutrients required by plants are abundant in nature, only a small amount of P is readily available in the form for plant uptake [59].

All tested *Trichoderma* isolates were found to produce siderophores that changed color from green to orange on the CAS agar plates (Figure 4). The fastest color change was observed in *T. spirale* HT009 (Figure 4b) at five DAI, while the other three *Trichoderma* isolates took 8–10 days. Siderophores are ferric ion-specific chelating agents formed by microorganisms to combat low iron stress [61]. In order to make the ferric ion [Fe(III)] available to microbials and plants, siderophores play a crucial role in chelating it from the surrounding environment [62]. According to Kobayashi and Nishiwaza [63], Fe is an essential micronutrient for plant growth. Many of the BCAs with significant antagonistic characteristics produce siderophores that chelate the available iron and prevent iron uptake by the respective pathogen which indirectly limits the proliferation and root colonization of the pathogen [64]. 

#### 3.2.5. Scoring of the Biochemical Properties Index

The scoring system presented in Table 10 was developed to summarize the biochemical assays performed by *Trichoderma* isolates for their antifungal activities, lytic enzyme production, and plant growth-promoting activity. *T. asperellum* ST011 ranked the most activity in all assays, except siderophore production, and a total score of 30 was recorded. *T. spirale* (HT009) performed high activity in all assays, except β-1,3 glucanase and IAA production. Based on the biochemical assays, the isolates *T. asperellum* ST011 and *T. spirale* HT009 were selected to perform in vivo assays against *R. microporus*.

### 3.3. Nursery Experiments

#### 3.3.1. Disease assessment

**Figure 5:**
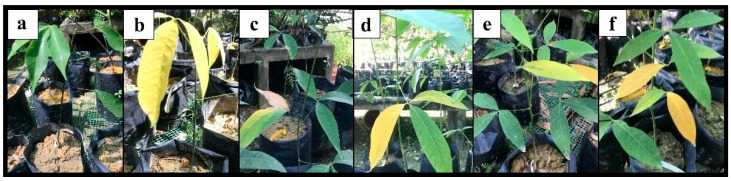
Examples of above-ground symptoms on rubber seedlings after six months of treatments with (**a**) T1, a plant without any pretreatment and Rigidoporus microporus inoculation (the negative control); (**b**) T2, a plant inoculated with Rigidoporus microporus but without any pretreatment (the positive control); (**c**) T3, a plant pretreated with Trichoderma asperellum and inoculated with Rigidoporus microporus; (**d**) T4, a plant pretreated with Trichoderma spirale and inoculated with Rigidoporus microporus; (**e**) T5, a plant pretreated with the combination Trichoderma asperellum + Trichoderma spirale and inoculated with Rigidoporus microporus; and (**f**) a T6, plant pretreated with propiconazole and inoculated with Rigidoporus microporus.

**Figure 6:**
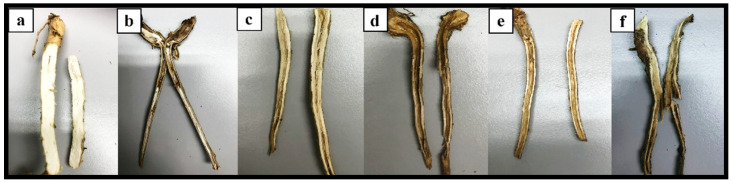
Examples of below-ground symptoms on rubber seedlings after six months of treatments with (**a**) T1, a plant without any pretreatment and Rigidoporus microporus inoculation (the negative control); (**b**) a T2, plant inoculated with Rigidoporus microporus but without any pretreatment (the positive control); (**c**) T3, a plant pretreated with Trichoderma asperellum and inoculated with Rigidoporus microporus; (**d**) T4, a plant pretreated with Trichoderma spirale and inoculated with Rigidoporus microporus; (**e**) T5, a plant pretreated with the combination (Trichoderma asperellum + Trichoderma spirale) and inoculated with Rigidoporus microporus; and (**f**) T6, a plant pretreated with propiconazole and inoculated with Rigidoporus microporus.

In this study, (-) the control plants of T1 without *R. microporus* inoculation showed no symptoms until the end of the experiment. Plants inoculated with *R. microporus*, (+) control plants of T2 (without pretreatment), plants pretreated with biocontrol suspensions (T3, T4, and T5), and plants pretreated with chemical fungicide (T6) showed above and below-ground symptoms of WRD at various degrees, i.e yellowing of the leaves followed by wilting and root rotting incidence (Figure 5 and Figure 6). Six months after inoculation (MAI) of *R. microporus*, (+) the control plants in T2 showed the greatest disease severity index of 68.54 based on the above- and below-ground scorings (Table 11). A lower disease severity index (DSI) value for T3, T4, T5, and T6 indicated that the disease suppression was effective with the biocontrol and chemical applications. The DSI of plants pretreated with the *Trichoderma* biocontrol suspension in T3, T4, and T5 was recorded at 28.44%, 42.71%, and 27.92%, respectively, after six MAI of *R. microporus* inoculation (Table 11). The investigation showed that biocontrol suspension of just *T. asperellum* alone and a combination of *T. asperellum* + *T. spirale* reflected higher efficacy against *R. microporus*, with their efficiency percentage recorded at 58.51% and 59.27%, respectively (Table 11). Thus, the biocontrol suspensions were effective, with a reduction of DSI by almost 40% compared to (+) the control T2. Despite the promising results shown by *Trichoderma* isolates, the application of *T. spirale* alone is less effective when compared to applying *T. asperellum* (T3) alone or with the combination of both *Trichoderma* suspensions (T5), suggesting that *T. asperellum* played a more important role in suppressing WRD in the present study. There was no significant difference between the DSI value in plants treated with just *T. asperellum* (T3) and the one with the combination of *T. asperellum* and *T. spirale* (T5).

On the other hand, the formation of rhizomorphs in the rubber seedlings of T6 (the treatment with chemical fungicide) was noticeably lesser when compared to (+) the control T2, with a DSI of 33.54% recorded. Despite the positive effect of propiconazole on the WRD development for the first three MAI, the rhizomorph formation was increasing gradually thereafter. The mycelia of *R. microporus* appeared to grow rapidly and spread again with the white rhizomorphs noticeable on the root surface.

As shown in Table 12, the mortality of the rubber seedlings clone RRIM600 revealed that the plants in T2 which served as (+) the control have caused serious damage, as the survival rate was only recorded at 33.33% in the six MAI of *R. microporus*. The greatest survival rate of 66.67% was achieved by plants treated with T3 and T5. This was followed by T6 and T4, with the survival rate of plants recorded being 58.33% and 50.00%, respectively. 

The present study demonstrated that *Trichoderma* isolates have the potential to improve soil health and compete with *R. microporus* when the host plants were challenged with the respective pathogen at which their applications were aimed to function as a protective approach. The nursery trial demonstrated that the rubber clone RRIM600 challenged with *R. microporus* without any biocontrol or chemical treatments had serious damage, with a mortality rate as high as 66.67%, which indicated that the rubber clone RRIM600 was susceptible to *R. microporus*. 

Prior application of the BCAs could not completely eliminate the presence of *R. microporus* rhizomorphs. It was observed that pretreated soil with selected *Trichoderma* isolates prior to the inoculation of *R. microporus* was only able to reduce the disease severity by 27–43%, as the infected plants were not completely free of *R. microporus* infection even after 60 days. Although, it was reported by Suryanto et al. [21] that BCAs work best as a preventive measure rather than a curative. Systematic inoculation of *Trichoderma* isolates in the soil even after infection may help to limit the further spread of *R. microporus* by securing *Trichoderma* as the dominating role in suppressing the pathogen.

#### 3.3.2. Transmission Electron Microscopy (TEM) Observation

**Figure 7:**
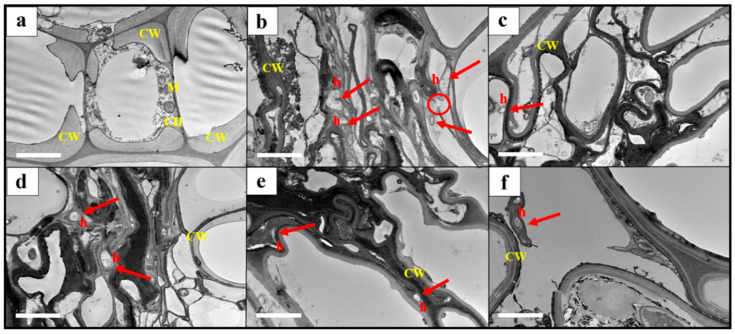
Examples of the TEM observation of root sections from rubber seedlings pretreated with *Trichoderma* suspensions and chemical fungicide after inoculation with *Rigidoporus microporus* for six months. (**a**) T1, a plant without any pretreatment and *Rigidoporus microporus* inoculum (as the negative control); (**b**) T2, a plant inoculated with *Rigidoporus microporus* but without any pretreatment (as the positive control); (**c**) T3, a plant pretreated with *Trichoderma asperellum* and inoculated with *Rigidoporus microporus*; (**d**) T4, a plant pretreated with *Trichoderma spirale* and inoculated with *Rigidoporus microporus*; (**e**) T5, a plant pretreated with a combination of *Trichoderma asperellum* + *Trichoderma spirale* and inoculated with *Rigidoporus microporus*; and (**f**) T6, a plant pretreated with chemical propiconazole and inoculated with *Rigidoporus microporus*. CW: cell wall; CH: chloroplast; M: mitochondrion, and h: hyphae of *Rigidoporus microporus* (arrow). (Scale bar = 2 µm; magnification: ×2500).

The TEM observation revealed that the root tissues of the healthy seedling in T1 were normal and free from *R. microporus,* with thick and smooth cell walls (Figure 7a). The results showed the presence of the pathogen hyphae penetrated the root tissues of the plant in T2 (Figure 7b). The cell walls of roots infected with *R. microporus* were extensively degraded and ruptured. Cells of the infected root were severely compressed compared to the healthy root in T1, which were still firm in shape. The penetration of *R. microporus* hyphae into the root tissues was noticeable in plants treated with *Trichoderma* suspension and fungicide (Figure 7c–f), yet a lesser amount of hyphae penetration was observed, particularly in T3, T5, and T6. Therefore, cell damage was reduced compared to T2 root tissue. Reduced penetration of *R. microporus* hyphae into host root systems may be related to the ability of the biocontrol agents, *Trichoderma* spp. in producing cell wall degrading enzymes acting on the hyphae of *R. microporus*, thus weakening the pathogen. Biocontrol suspensions of *T. asperellum* and *T. spirale* were effective in slowing disease progression, which is believed to have reduced disease severity.

## 4. Conclusions

In this study, a total of 35 fungal isolates were successfully established from the rubber tree rhizosphere soil and further screened for their antagonistic potential against *R. microporus* in dual culture plates. Four *Trichoderma* isolates represented by *T. asperellum*, *T. koningiopsis*, *T. spirale,* and *T. reesei* showed high antagonistic potential in the inhibition of radial growth of *R. microporus* of 75% or more. In vitro results clearly demonstrated that *T. asperellum* and *T. spirale* isolates showed high antifungal activity in reducing mycelia growth of the pathogen *R. microporus* through volatile and non-volatile productions. Moreover, the screening of enzymes revealed their capability to excrete extracellular lytic enzymes, such as chitinase, cellulase, and glucanase, at different levels. Positive results demonstrated by the *Trichoderma* isolates in the plant growth-promoting activities, such as indole acetic acid, siderophores productions, and phosphate solubilization have further supported the use of selected *Trichoderma* isolates against *R. microporus* in vivo. In nursery assessments, studies have shown that soil pretreated with *T. asperellum* ST013 as a single treatment or combination with *T. spirale* HT009 was able to reduce the disease severity index of *R. microporus* to a similar extent as in the propiconazole chemical treatment. Further studies should focus on the potential adverse effects of *T. asperellum* ST011 by assessing the knowledge of its environmental fate and behavior in terms of persistence, survival, and dispersion in different soil types.

## Figures and Tables

**Table 1:** Type of treatments on rubber seedlings using *Trichoderma* suspensions and chemical fungicide for white root rot disease assessment in the nursery.

Treatment	Description
T1	Tree without inoculation of *R. microporus*
T2	Tree inoculated with *R. microporus* and without application of biocontrol suspension or chemical fungicide
T3	Biocontrol application with 100 mL of 10^8^ cfu/mL suspension of *T. asperellum* ST011 to the planting media once a week for a month before the tree is inoculated with an *R. microporus* wood block
T4	Biocontrol application with 100 mL of 10^8^ cfu/mL suspension of *T. spirale* HT009 to the planting media once a week for a month before the tree is inoculated with an *R. microporus* wood block
T5	Biocontrol application with 100 mL of 10^8^ cfu/mL suspension of *T. asperellum* ST011 + *T. spirale* HT009 to the planting media once a week for a month before the tree is inoculated with an *R. microporus* wood block
T6	Chemical application with 100 mL Kentil propiconazole 250 EC in a diluted form of 1% to the planting media once a week for a month before the tree is inoculated with an *R. microporus* wood block

**Table 2:** Assessment of the disease severity index based on the justifications of above- and below-ground symptoms.

Scoring	Above-Ground Symptom	Below-Ground Symptom
0	Healthy with green leaves	Fungal infections not found
1	1–25% of yellowing leaves	Fungal infections in the root are less than 1%
2	26–50% of wilted leaves	1–10% of fungal infections in the root
3	51–75% defoliation	11–50% of fungal infections in the root
4	76–100% death of the plant	51–90% of fungal infection in the root
5	-	Fungal infections in the root of more than 90%

(Source: Wattanasilakorn et al. [43]).

**Table 3:** Fungal community associated with the soil sampled in this study from a rubber plantation at the Rubber Research Institute of Malaysia and its identity percentage from the NCBI website.

Isolate ID	Fungal Identity	Genbank Accession Number	Genbank Accession Number (% Identity)
HT001	*Trichoderma koningiopsis*	MH512944	EU718083 (100%)
HT002	*Talaromyces* sp.	MH512945	KY643686 (99%)
HT003	*Purpureocillium lilacinum*	MH512946	KX641490 (99.83%)
HT004	*Fusarium oxysporum*	MH512947	MG020429 (100%)
HT005	*Talaromyces aculeatum*	MH512948	KM458839 (99.83%)
HT006	*Talaromyces aculeatum*	MH512949	KM458839 (99.83%)
HT007	*Cunninghamella bainieri*	MH512950	KF201293 (99.86%)
HT008	*Metarhizium anisopliae*	MH512951	KX806656 (99.82%)
HT009	*Trichoderma spirale*	MH512952	JF439515 (99.67%)
HT010	*Penicillium* sp.	MH512953	KM066554 (100%)
HT011	*Purpureocillium lilacinum*	MH512954	KC478538 (100%)
HT012	*Penicillium* sp.	MH512955	KM066554 (100%)
HT013	*Clonostachys* sp.	MH512956	KY419566 (99.42%)
HT014	*Scedosporium boydii*	MH512957	KP132695 (99.35%)
HT015	*Trichoderma koningiopsis*	MH512958	EU718083 (100%)
HT016	*Aspergillus nomius*	MH512959	AB828718 (100%)
HT017	*Penicillium* sp.	MH512960	HM469409 (100%)
HT018	*Byssochlamys spectabilis*	MH512961	KC311513 (99.51%)
ST001	*Gongronella butleri*	MN103605	KM246758 (97.95%)
ST003	*Penicillium* sp.	MH512962	KM066554 (99.49%)
ST004	*Trichoderma spirale*	MH512963	JF439515 (99.83%)
ST005	*Fusarium oxysporum*	MH512964	MG020429 (100%)
ST006	*Trichoderma spirale*	MH512965	HQ229947 (100%)
ST007	*Xepicula leucotricha*	MH512966	KU847251 (97.94%)
ST008	*Penicillium* sp.	MH512967	KY425719 (100%)
ST009	*Trichosporiella* sp.	MH512968	DQ069049 (96.43%)
ST010	*Lasiodiplodia theobromae*	MH512969	KR260793 (100%)
ST011	*Trichoderma asperellum*	MH512970	MG657259 (100%)
ST012	*Arcopilus cupreus*	MH512971	MF599416 (97.61%)
ST013	*Trichoderma reesei*	MH512972	MH047196 (99.37%)
ST014	*Trichoderma koningiopsis*	MH512973	EU718083 (100%)
ST015	*Wiesneriomyces laurinus*	MH512974	KP057801 (86.12%)
ST016	*Penicillium singorense*	MH512975	LT558940 (100%)
ST017	*Byssochlamys spectabilis*	MH512976	KC254066 (100%)
ST018	*Clonostachys* sp.	MH512977	KX343160 (98.25%)

* http://www.ncbi.nlm.nih.gov/BLAST, accessed 29 October 2021.

**Table 4:** Effects of 35 isolates of soil-borne fungi in a dual culture assay on *Rigidoporus microporus* radial growth, percentage inhibition of radial growth (PIRG), and Bell’s ranking scale [26] categories after 5 days of culture in Petri dishes with the potato dextrose agar.

No.	Isolate ID	Fungal Identity	Radial Growth (mm) *	PIRG (%) *	Bell’s Ranking Scale Categories
1	HT001	*Trichoderma koningiopsis*	6.63 ± 1.10 ^bcd^	76.94 ± 3.84 ^cd^	R1
2	HT002	*Talaromyces* sp.	19.37 ± 1.06 ^jklm^	32.60 ± 3.67 ^ijkl^	R4
3	HT003	*Purpureocillium lilacinum*	15.94 ± 0.60 ^hij^	44.55 ± 2.08 ^ghi^	R5
4	HT004	*Fusarium oxysporum*	16.00 ± 1.15 ^hij^	44.35 ± 4.00 ^ghi^	R4
5	HT005	*Talaromyces aculeatum*	23.13 ± 0.70 ^mno^	19.52 ± 2.45 ^lmn^	R3
6	HT006	*Talaromyces aculeatum*	21.70 ± 2.25 ^lmn^	24.51 ± 7.83 ^klm^	R4
7	HT007	*Cunninghamella bainieri*	1.96 ± 0.29 ^a^	93.18 ± 1.02 ^a^	R1
8	HT008	*Metarhizium anisopliae*	25.19 ± 1.40 ^no^	12.36 ± 4.88 ^mn^	R3
9	HT009	*Trichoderma spirale*	5.88 ± 1.47 ^ab^	79.55 ± 5.12 ^bc^	R2
10	HT010	*Penicillium* sp.	19.03 ± 1.92 ^jkl^	33.78 ± 6.69 ^ijk^	R3
11	HT011	*Purpureocillium lilacinum*	16.82 ± 0.94 ^ijk^	41.48 ± 3.29 ^hij^	R3
12	HT012	*Penicillium* sp.	16.17 ± 1.92 ^hijk^	43.75 ± 6.68 ^ghi^	R3
13	HT013	*Clonostachys* sp.	30.23 ± 1.79 ^qr^	nil	R4
14	HT014	*Scedosporium boydii*	33.48 ± 1.62 ^qr^	nil	R5
15	HT015	*Trichoderma koningiopsis*	11.87 ± 0.77 ^efg^	58.72 ± 2.67 ^ef^	R2
16	HT016	*Aspergillus nomius*	8.67 ± 1.65 ^bcdef^	69.83 ± 5.72 ^cde^	R1
17	HT017	*Penicillium* sp.	12.68 ± 2.23 ^fgh^	55.90 ± 7.77 ^fg^	R2
18	HT018	*Byssochlamys spectabilis*	13.74 ± 1.75 ^ghi^	52.18 ± 6.10 ^fgh^	R2
19	ST001	*Gongronella butleri*	26.11 ± 0.75 ^op^	9.15 ± 2.60 ^n^	R3
20	ST003	*Penicillium* sp.	9.92 ± 1.52 ^cdefg^	65.49 ± 5.27 ^def^	R3
21	ST004	*Trichoderma spirale*	12.28 ± 1.64 ^efgh^	57.27 ± 5.69 ^efg^	R2
22	ST005	*Fusarium oxysporum*	13.13 ± 0.74 ^ghi^	54.33 ± 2.58 ^fgh^	R4
23	ST006	*Trichoderma spirale*	12.29 ± 1.50 ^efgh^	57.25 ± 5.23 ^efg^	R2
24	ST007	*Xepicula leucotricha*	29.87 ± 1.34 ^pqr^	nil	R5
25	ST008	*Penicillium* sp.	20.09 ± 1.28 ^klm^	30.11 ± 4.46 ^jkl^	R4
26	ST009	*Trichosporiella* sp.	33.80 ± 1.98 ^r^	nil	R5
27	ST010	*Lasiodiplodia theobromae*	5.41 ± 1.78 ^ab^	81.19 ± 6.20 ^abc^	R1
28	ST011	*Trichoderma asperellum*	2.01 ± 1.63 ^a^	93.02 ± 5.68 ^ab^	R1
29	ST012	*Arcopilus cupreus*	19.12 ± 0.91 ^jklm^	33.47 ± 3.15 ^ijk^	R5
30	ST013	*Trichoderma reesei*	10.59 ± 0.70 ^defg^	63.14 ± 2.43 ^n^	R2
31	ST014	*Trichoderma koningiopsis*	6.53 ± 1.53 ^bc^	77.28 ± 5.33 ^cd^	R1
32	ST015	*Wiesneriomyces laurinus*	29.46 ± 1.38 ^pq^	nil	R5
33	ST016	*Penicillium singorense*	21.98 ± 1.93 ^lmn^	23.52 ± 6.72 ^klm^	R3
34	ST017	*Byssochlamys spectabilis*	8.52 ± 1.75 ^bcde^	70.35 ± 6.10 ^cde^	R1
35	ST018	*Clonostachys* sp.	31.41 ± 0.65 ^qr^	nil	R5
36	-	Control	28.74 ± 1.49	-	-

* Values are the mean of four replicates ± SD. Within each column, means with different letters are significantly different according to the Tukey’s multiple comparison test (*p* < 0.05). nil: no inhibition activity.

**Table 5:** Effects of three different concentrations of culture filtrate containing non-volatile metabolites produced by four *Trichoderma* isolates on *Rigidoporus microporus* radial growth and the percentage inhibition of radial growth (PIRG) after 5 days of growth at 28 ± 2 °C, in the dark.

Isolate (ID)	Concentration of Culture Filtrate (%)	Radial Growth (mm) *	PIRG (%) *
*T. koningiopsis* (HT001)	75	50.90 ± 1.21 ^d^	10.20 ± 2.14 ^d^
	50	60.01 ± 1.24 ^e^	nil
	25	64.01 ± 1.66 ^fg^	nil
*T. spirale* (HT009)	75	17.01 ± 1.49 ^b^	70.00 ± 2.63 ^b^
	50	28.51 ± 2.08 ^c^	49.71 ± 3.66 ^c^
	25	51.98 ± 0.95 ^d^	8.30 ± 1.68 ^d^
*T. asperellum* (ST011)	75	12.02 ± 1.74 ^a^	78.80 ± 3.08 ^a^
	50	12.81 ± 0.63 ^a^	77.41 ± 1.10 ^a^
	25	19.54 ± 0.99 ^b^	65.54 ± 1.75 ^b^
*T. reesei* (ST013)	75	53.34 ± 1.89 ^d^	5.90 ± 3.32 ^d^
	50	63.62 ± 0.97 ^f^	nil
	25	67.04 ± 0.57 ^g^	nil
Control	-	56.69 ± 2.31	-

* Values are the mean of four replicates ± SD. Within each column, means with different letters are significantly different according to Tukey’s multiple comparison test (*p* < 0.05). nil: no inhibition activity.

**Table 6:** Effects of volatile organic compounds produced by four *Trichoderma* isolates on *Rigidoporus microporus* radial growth and the percentage inhibition of radial growth (PIRG) using the overlapping plate assay after 5 days of growth at 28 ± 2 °C, in the dark.

Isolate (ID)	Radial Growth (mm) *	PIRG (%) *
*T. koningiopsis* (HT001)	45.61 ± 1.08 ^c^	33.42 ± 1.93 ^b^
*T. spirale* (HT009)	42.63 ± 0.59 ^b^	37.77 ± 1.05 ^b^
*T. asperellum* (ST011)	30.30 ± 1.15 ^a^	55.77 ± 2.06 ^a^
*T. reesei* (ST013)	50.17 ± 0.81 ^d^	26.76 ± 1.44 ^c^
Control	68.51 ± 0.31	-

* Values are the mean of four replicates ± SD. Within each column, means with different letters are significantly different according to Tukey’s multiple comparison test (*p* < 0.05).

**Table 7:** The main compounds identified in the methanol extracts of *Trichoderma* isolates by GC-MS, each having a >1% peak area and a ≥50% match quality in the NIST-17 library search.

Isolate (ID)	Retention Time	Probability	Compound Name	Molecular Formula	Area %	Compound Class
*T. koningiopsis (HT001)*	16.10	75.15	Benzeneacetic acid,4-hydroxy-	C_8_H_8_O_3_	1.24	Acid
	19.92	95.73	Benzenepropanoic acid,3,5-bis(1,1-dimethylethyl)-4-hydroxy-,methyl ester	C_18_H_28_O_3_	13.25	Ester
	27.56	76.20	13-Docosenamide,(Z)-	C_22_H_43_NO	24.30	Amide
	27.98	66.20	Dodecanoic acid,1,2,3-propanetriyl ester	C_39_H_74_O_6_	2.51	Acid
*T. spirale* (HT009)	19.92	91.88	Benzenepropanoic acid,3,5-bis(1,1-dimethylethyl)-4-hydroxy-,methyl ester	C_18_H_28_O_3_	9.95	Ester
	20.20	61.42	3,5-di-tert-Butyl-4-hydroxyphenylpropionic acid	C_17_H_26_O_3_	3.65	Acid
	27.52	79.42	13-Docosenamide,(Z)-	C_22_H_43_NO	32.32	Amide
	38.57	59.62	(5á)Pregnane-3,20á-diol, 14à,18à-[4-methyl-3-oxo-(1-oxa-4-azabutane-1,4-diyl)]-,diacetate	C_28_H_43_NO_6_	1.38	Ester
*T. asperellum* (ST011)	13.94	68.67	2-(4-Methoxyphenyl) ethanol	C_9_H_12_O_2_	5.69	Alcohol
	14.66	69.16	Benzeneethanol,4-hydroxy-	C_8_H_10_O_2_	18.42	Alcohol
	15.73	51.90	2,4-Di-tert-butylphenol	C_14_H_22_O	2.07	Phenol
	16.14	71.40	Benzeneacetic acid,4-hydroxy-	C_8_H_8_O_3_	1.53	Acid
	27.52	81.40	13-Docosenamide,(Z)-	C_22_H_43_NO	11.98	Amide
	34.53	57.36	Dodecanoic acid,1,2,3-propanetriyl ester	C_39_H_74_O_6_	8.59	Acid
	36.20	50.14	1-Dodecanoyl-3-myristoyl glycerol	C_29_H_56_O_5_	5.68	Acid
	36.49	54.14	1-Dodecanoyl-3-myristoyl glycerol	C_29_H_56_O_5_	3.07	Acid
*T. reesei* (ST013)	12.56	59.24	4-Ketopimelic	C_7_H_10_O_5_	1.36	Acid
	14.62	70.29	Benzeneethanol,4-hydroxy-	C_8_H_10_O_2_	2.08	Alcohol
	15.73	50.67	2,4-Di-tert-butylphenol	C_14_H_22_O	3.25	Phenol
	18.13	62.84	11,13-Dihydroxy-tetradec-5-ynoic acid, methyl ester	C_15_H_26_O_4_	2.53	Ester
	19.92	96.59	Benzenepropanoic acid, 3,5-bis(1,1-dimethylethyl)-4-hydroxy-,methyl ester	C_18_H_28_O_3_	17.11	Ester
	27.57	83.99	13-Docosenamide, (Z)-	C_22_H_43_NO	36.05	Amide

**Table 8:** Enzyme activities (μmol/min) of four *Trichoderma* isolates in liquid cultures (see Section 2.4.2 for media composition and growth conditions).

Isolate (ID)	Chitinase	Cellulase	β-1,3 Glucanase
*T. koningiopsis* (HT001)	0.02 ± 0.00 ^b^	0.11 ± 0.01 ^c^	0.03 ± 0.00 ^b^
*T. spirale* (HT009)	0.02 ± 0.00 ^b^	0.18 ± 0.01 ^b^	nil
*T. asperellum* (ST011)	0.04 ± 0.00 ^a^	0.26 ± 0.03 ^a^	0.06 ± 0.00 ^a^
*T. reesei* (ST013)	0.02 ± 0.00 ^b^	0.04 ± 0.01 ^d^	0.02 ± 0.00 ^c^

* Values are the mean of four replicates ± SD. Within each column, means with different letters are significantly different according to Tukey’s multiple comparison test (*p* < 0.05). nil: no activity observed.

**Table 9:** Indole acetic acid (IAA) produced by *Trichoderma* isolates, *T. koningiopsis* HT001, *T. spirale* HT009, *T. asperellum* ST011, and *T. reesei* ST013.

Isolate (ID)	IAA Production (µg/mL) *
*T. koningiopsis* (HT001)	nil
*T. spirale* (HT009)	nil
*T. asperellum* (ST011)	1.68 ± 0.10
*T. reesei* (ST013)	0.83 ± 0.05

* Values are the mean of four replicates ± SD. nil: no activity observed.

**Table 10:** Scoring of the biochemical assays performed by *Trichoderma* isolates.

Activity		Isolate (ID) *			
		*T. koningiopsis* (HT001)	*T. spirale* (HT009)	*T. asperellum* (ST011)	*T. reesei* (ST013)
Antibiosis	Non-volatile metabolite	2	3	4	2
	Volatile metabolite	3	3	4	1
Hydrolytic enzyme	Chitinase	3	3	4	3
	Cellulase	2	3	4	1
	β-1,3 glucanase	3	0	4	2
Competition	Phosphate solubilization	0	4	4	2
	IAA production	0	0	4	3
	Siderophore production	1	4	2	3
**Total score**		**14**	**20**	**30**	**17**

* Using a scale from 0 = isolate showing no activity to 4 = isolate showing the highest activity.

**Table 11:** Disease severity index (DSI) of rubber seedlings based on above- and below-ground symptoms and the efficacy of *Trichoderma* suspensions (*Trichoderma asperellum* + *Trichoderma spirale*) or propiconazole fungicide in the suppression of white root rot disease caused by *Rigidoporus microporus*.

Treatment	DSI (%)		Average DSI (%) *	Efficacy (%)
	Above-Ground (Foliar Discoloration)	Below-Ground (Root Rotting)		
T2	52.08	85.00	68.54 ± 23.28	-
T3	21.88	35.00	28.44 ± 9.28	58.51
T4	35.42	50.00	42.71 ± 10.31	37.69
T5	20.83	35.00	27.92 ± 10.02	59.27
T6	27.08	40.00	33.54 ± 9.13	51.06

* Note: Data are the means ± SD of four replicates with six plants per treatment in each replicate.

**Table 12:** Mortality rate per month and survival rate of rubber seedlings pretreated with *Trichoderma* suspensions (*Trichoderma asperellum* + *Trichoderma spirale*) or propiconazole after inoculation with *Rigidoporus microporus* for six months.

Treatment	Mortality Rate per Month (%)						Survival Rate at 6 MAI (%) *
	1	2	3	4	5	6	
T2	0.00	12.50	20.83	16.67	8.33	8.33	33.33 ± 7.30
T3	0.00	4.17	8.33	4.17	8.33	8.33	66.67 ± 3.40
T4	0.00	8.33	12.50	8.33	12.50	8.33	50.00 ± 4.56
T5	0.00	4.17	4.17	8.33	8.33	8.33	66.67 ± 3.48
T6	0.00	4.17	8.33	8.33	12.50	8.33	58.33 ± 3.96

* Note: Data are the means ± SD of four replicates with six plants per treatment in each replicate. MAI: month after inoculation of *Rigidoporus microporus.*

## Data Availability

All data generated or analyzed are included in this article.
